# Distinct ALK Expression Patterns Are Associated with Canonical and Noncanonical *STRN::ALK* Transcript Architectures in Oncocytic Thyroid Neoplasms

**DOI:** 10.1007/s12022-026-09924-0

**Published:** 2026-07-02

**Authors:** Debora Mota Dias Thomaz, Thais Biude Mendes, Thaise Nayane Ribeiro Carneiro, Luiza Sisdelli, Ana Carolina de Jesus Paniza, André Uchimura Bastos, Janete Maria Cerutti

**Affiliations:** 1 Genetic Bases of Thyroid Tumors Laboratory, Division of Genetics, Department of Morphology and Genetics, Universidade Federal de São Paulo - Escola Paulista de Medicina, Pedro de Toledo 669, 11th floor, São Paulo, SP 04039-032 Brazil; 2 Department of Genetics, Perelman School of Medicine, University of Pennsylvania, Philadelphia, USA

**Keywords:** Oncocytic Thyroid Neoplasms, Anaplastic Lymphoma Kinase, ALK Fusion Proteins, Immunohistochemistry, Fluorescence In Situ Hybridization.

## Abstract

Oncocytic thyroid carcinomas are characterized by a unique chromosomal landscape and frequent resistance to radioactive iodine therapy, underscoring the need for improved molecular characterization. Although ALK rearrangements, particularly *STRN::ALK* fusions, have been described in thyroid carcinomas, their biological and clinical significance in oncocytic thyroid neoplasms remains unclear. This study investigated *STRN* exon 3-*ALK* exon 20-derived transcripts in 56 oncocytic thyroid neoplasms using RT-PCR, followed by cloning and Sanger sequencing of positive cases. Transcript-positive cases were further evaluated by fluorescence in situ hybridization (FISH), immunohistochemistry (IHC), and in silico structural modeling. *STRN*::*ALK*-derived transcripts were detected in 9 of 56 tumors; however, their frequency reflected the combined detection of distinct transcript architectures. Only 2 of 56 tumors (3.6%) harbored canonical in-frame *STRN*::*ALK* fusions, whereas 7 of 56 tumors (12.5%) contained noncanonical out-of-frame variants. Although ALK rearrangement by FISH was confirmed in all transcript-positive tumors irrespective of transcript architecture, detectable ALK protein expression by IHC was observed only in tumors harboring canonical in-frame fusions, whereas all noncanonical variants lacked detectable ALK protein expression. Structural modeling was concordant with these observations, suggesting preservation of kinase-domain features in canonical fusions and predicted disruption in noncanonical variants. Overall, *STRN::ALK* transcripts in oncocytic thyroid neoplasms exhibit marked architectural heterogeneity with distinct ALK protein expression patterns. ALK FISH-positive/IHC-negative cases should be interpreted cautiously, as they may not represent biologically equivalent ALK-driven neoplasia, with uncertain therapeutic relevance. Integrated molecular and protein-level assessment may support interpretation of ALK alterations in oncocytic thyroid neoplasms.

**Supplementary Information:**

The online version contains supplementary material available at 10.1007/s12022-026-09924-0.

## Introduction

Thyroid cancer is the most common endocrine malignancy, and its incidence continues to rise worldwide [1]. Among its histological subtypes, oncocytic carcinoma of the thyroid (OCA), formerly known as Hürthle cell carcinoma, accounts for 2–5% of cases [2]. Once considered a subtype of follicular thyroid carcinoma (FTC), OCA is now recognized as a distinct clinicopathological entity in the current World Health Organization (WHO) classification of thyroid neoplasms [3, 4].

Histologically, OCA is defined as an invasive malignant follicular cell neoplasm composed of at least 75% oncocytic cells, lacking the nuclear features of papillary thyroid carcinoma and high-grade characteristics [2, 5–8]. It is distinguished from benign oncocytic adenoma of the thyroid (OA) by the presence of capsular and/or vascular invasion, whereas OA remains encapsulated and lacks invasive features [1].

Beyond its morphologic features, OCA exhibits a distinctive genomic profile characterized by widespread chromosomal losses and recurrent mitochondrial dysfunction. Homoplasmic mtDNA mutations, typically affecting Complex I of the respiratory chain, frequently co-exist with extensive chromosomal loss of heterozygosity (LOH), genome haploidisation-type copy number alterations, uniparental disomy, and chromosomal endoreduplication [2, 5, 6, 8–12]. This highly unstable genomic landscape may facilitate the emergence of structurally diverse genomic rearrangements, including potentially recurrent but biologically heterogeneous fusion events.

Clinically, a subset of these tumors demonstrates more aggressive behavior and reduced iodine avidity, thereby limiting the effectiveness of radioactive iodine (RAI) therapy [2, 7, 8]. Collectively, these features highlight the need for deeper molecular characterization with potential diagnostic and therapeutic relevance.

In this context, gene fusions involving receptor tyrosine kinases (RTKs) have emerged as clinically relevant oncogenic drivers in thyroid cancer, with implications for diagnosis, prognosis, and targeted therapy [13, 14]. Among these, rearrangements involving the anaplastic lymphoma kinase (ALK) gene have been described across several thyroid cancer subtypes, particularly papillary, poorly differentiated, and anaplastic thyroid carcinomas [15–22].

In thyroid neoplasms, *STRN* is the most frequently reported *ALK* fusion partner, most commonly involving the canonical in-frame fusion between *STRN* exon 3 and *ALK* exon 20. These *STRN::ALK* rearrangements are typically associated with detectable ALK protein expression and clinical responsiveness to ALK-directed therapies, underscoring their therapeutic relevance [15, 16, 18, 23–25]. Although isolated cases of ALK-rearranged oncocytic thyroid neoplasms have previously been reported, *STRN::ALK* alterations in a dedicated oncocytic cohort have not been systematically investigated [26, 27].

Here, we evaluated *STRN* exon 3-*ALK* exon 20-derived transcripts in a dedicated cohort of oncocytic thyroid neoplasms using an integrated approach combining transcript-based detection, Sanger sequencing, fluorescence in situ hybridization (FISH), immunohistochemistry (IHC), and in silico structural modeling. Our findings reveal heterogeneity in *STRN*::*ALK* transcript architecture among oncocytic thyroid neoplasms and suggest that, although genomic ALK rearrangement can be confirmed by FISH across distinct transcript variants, only canonical in-frame fusions are associated with detectable ALK protein expression and predicted preservation of kinase-domain features.

## Materials and Methods

### Study Cohort

This single-institution retrospective study included consecutive formalin-fixed, paraffin-embedded (FFPE) thyroid specimens from patients who underwent thyroidectomy at Hospital São Paulo, Universidade Federal de São Paulo (UNIFESP), between 1995 and 2015, with diagnosis of oncocytic thyroid carcinomas (OCA) or oncocytic thyroid adenomas (OA). All cases were initially diagnosed at the Department of Pathology, UNIFESP, and were subsequently reviewed and reclassified by an expert thyroid pathologist (ACJP) according to the diagnostic criteria established in the 2022 WHO Classification of Thyroid Tumors [3, 4]. To ensure histological representativeness, superficial and deeper sections from each block (approximately 100 μm apart) were evaluated on hematoxylin-and eosin-stained slides to confirm diagnosis and adequate tumor content. Representative FFPE blocks containing > 70% tumor cellularity were selected for molecular analyses. In cases not meeting this threshold due to the presence of adjacent non-neoplastic thyroid tissue, tumor-rich areas identified by the reviewing pathologist were manually macrodissected prior to nucleic acid extraction to enrich for neoplastic cells. The final cohort comprised 27 OCA and 29 OA cases. The study was conducted in accordance with the Brazilian National Research Ethics Committee (CAAE: 56882116.7.0000.550). Detailed clinicopathological characteristics are summarized in Table 1.

**Table 1:** Clinical and pathological characteristics of all oncocytic thyroid tumors

Case ID	Diagnosis	Age at Diagnosis (years)	Sex	Tumor size (cm)	Capsular invasion	Vascular invasion	WHO 2022 tumor subtype	TNM
1	OCA	62	F	2	N	Y	EA	pT2NxMx
2	OCA	51	M	2.2	Y	Y	EA	pT2NxMx
3	OCA	55	F	0.3	N	Y	EA	pT1aNxMx
4*	OCA	75	F	2.6	Y	Y	WI	pT2NxMx
5*	OCA	58	F	1.4	Y	N	MI	pT1bNxMx
6	OCA	47	F	1.8	N	Y	EA	pT1bNxMx
7*	OCA	48	F	1.8	N	Y	EA	pT1bNxMx
8	OCA	86	F	5.5	N	Y	EA	pT3NxMx
9*	OCA	59	F	2	Y	Y	EA	pT2NxMx
10*	OCA	28	F	4	N	Y	EA	pT2NxMx
11*	OCA	31	F	2.5	N	Y	EA	pT2NxMx
12*	OCA	71	F	1.2	Y	N	MI	pT1bNxMx
13	OCA	58	F	4	Y	N	MI	pT3NxMx
14	OCA	53	M	4	Y	Y	EA	pT3NxMx
15	OCA	44	M	4.5	N	Y	EA	pT3NxMx
16	OCA	40	F	2	Y	Y	WI	pT2NxMx
17	OCA	37	F	3	Y	Y	EA	pT2NxMx
18	OCA	53	F	2.5	Y	N	MI	pT2NxMx
19	OCA	65	F	4.5	Y	Y	EA	pT3NxMx
20	OCA	82	F	10	Y	Y	EA	pT4aNxMx
21	OCA	64	F	2.1	Y	Y	WI	pT2NxMx
22	OCA	51	M	2.1	N	Y	EA	pT2NxMx
23	OCA	40	F	0.5	Y	Y	WI	pT1aNxMx
24	OCA	72	M	3.2	N	Y	EA	pT3NxMx
25	OCA	54	F	3	Y	Y	EA	pT2NxMx
26	OCA	68	F	10	Y	Y	EA	pT3N0Mx
27	OCA	59	F	0.5	Y	Y	EA	pT1aNxMx
28*	OA	67	F	3	N	N	NA	NA
29	OA	51	M	2.2	N	N	NA	NA
30	OA	48	F	1	N	N	NA	NA
31	OA	63	F	1.8	N	N	NA	NA
32	OA	42	F	4	N	N	NA	NA
33	OA	39	F	1	N	N	NA	NA
34	OA	44	F	4	N	N	NA	NA
35	OA	NR	F	3.5	N	N	NA	NA
36	OA	27	F	2	N	N	NA	NA
37	OA	NR	F	4	N	N	NA	NA
38	OA	53	M	2	N	N	NA	NA
39	OA	41	F	2.5	N	N	NA	NA
40*	OA	50	F	4	N	N	NA	NA
41	OA	68	F	NR	N	N	NA	NA
42	OA	NR	F	3.5	N	N	NA	NA
43	OA	79	F	12	N	N	NA	NA
44	OA	62	F	2.2	N	N	NA	NA
45	OA	43	F	2	N	N	NA	NA
46	OA	66	F	3.5	N	N	NA	NA
47	OA	73	F	4.5	N	N	NA	NA
48	OA	35	F	2.5	N	N	NA	NA
49	OA	53	F	6.5	N	N	NA	NA
50	OA	64	F	0.8	N	N	NA	NA
51	OA	29	F	1.8	N	N	NA	NA
52	OA	64	M	1.8	N	N	NA	NA
53	OA	23	F	5.5	N	N	NA	NA
54	OA	66	F	2.1	N	N	NA	NA
55	OA	51	F	0.7	N	N	NA	NA
56	OA	27	F	2.5	N	N	NA	NA

*STRN::ALK fusion positive cases; *OCA* Oncocytic Carcinoma of the Thyroid, *OA* Oncocytic Adenoma of the Thyroid, *F* Female, *M* Male, *NR* Not Reported, *Y* Yes, *N* No, *MI* Minimally invasive, *WI* Widely invasive, *EA* Encapsulated angioinvasive, *NA* Not Applicable, pTNM staging was applied only to malignant tumors according to AJCC criteria

### *STRN::ALK* Fusion Transcript Screening and Characterization

Total RNA was extracted from three 10-µm FFPE sections using the RecoverAll™ Total Nucleic Acid Isolation Kit (Applied Biosystems, Waltham, MA, USA), following the manufacturer’s protocol. RNA quantity and purity were assessed using a NanoDrop ND-2000 spectrophotometer (Thermo Fisher Scientific, Waltham, MA, USA). For cDNA synthesis, 500 ng of total RNA was treated with DNase and reverse-transcribed using the SuperScript™ III First-Strand Synthesis System (Invitrogen, Waltham, MA, USA) with a combination of oligo(dT)20 primers and random hexamers, according to the manufacturer’s instructions. cDNA integrity and amplifiability were evaluated by PCR amplification of the housekeeping gene *RPS8* (125 bp), as previously described [28]. All 56 cases yielded amplifiable cDNA and passed quality control criteria.

Screening for *STRN::ALK* fusion transcripts was performed by conventional PCR using primers targeting *STRN* exon 3 (forward: 5’-CGGGACAGAATTGAATCAGG-3’) and *ALK* exon 20 (reverse: 5’-CAAGCCATGCAGATGGAGC-3’). PCR amplifications were carried out in a final volume of 50 µL containing 2 µL of cDNA, 0.5 U of Platinum™ Taq DNA Polymerase, 1× PCR buffer, 1.5 mM MgCl2, 200 µM dNTPs, and 5 pmol of each primer (Invitrogen). Cycling conditions included an initial denaturation at 95 °C for 5 min, followed by 40 cycles of 95 °C for 30 s, 58 °C for 30 s, and 72 °C for 30 s, with a final extension at 72 °C for 5 min. The canonical *STRN* exon 3-*ALK* exon 20 amplicon was expected to generate a 94 bp product. PCR products were resolved on 2.5% agarose gels and visualized using a Gel Doc™ EZ Imaging System (Bio-Rad Laboratories, Hercules, CA, USA).

To validate fusion transcripts and map breakpoint sequences, each RT-PCR-positive case underwent three independent PCR amplifications. Amplification products were independently cloned into the pCR™2.1-TOPO^®^ vector using the TOPO TA Cloning Kit (Thermo Fisher Scientific) according to the manufacturer’s instructions. From each independent cloning reaction, one bacterial colony was randomly selected, expanded, and subjected to Sanger sequencing using the BigDye™ Terminator Cycle Sequencing Kit (Thermo Fisher Scientific), as previously described [29], yielding three independently derived sequences per case. This approach enabled assessment of reproducibility across independent amplification and cloning events but did not permit evaluation of intratumoral transcript heterogeneity or low-frequency subclonal variants.

To assess whether recurrent noncanonical *STRN::ALK* junctions could reflect sequence-dependent RT-PCR artifacts, the nucleotide sequences flanking each breakpoint were manually inspected for features potentially associated with polymerase slippage or template switching, including microhomology, direct repeats, low-complexity regions, GC-rich motifs, and predicted secondary structure-prone sequences.

### *ALK* Break-Apart Fluorescence in situ hybridization (FISH)

All nine RT-PCR-positive cases were further evaluated for *ALK* rearrangements using a dual-color break-apart. Analyses were performed on 3-µm FFPE sections using locus-specific bacterial artificial chromosome (BAC) probes targeting *ALK* (2p23.2; RP11-418E15 and RP11-203K5; Invitrogen), labeled with Spectrum Green and Spectrum Red by nick translation (Abbott Molecular, Chicago, IL, USA), according to the manufacturer’s instructions.

Sections were deparaffinized, rehydrated, and subjected to pretreatment, followed by denaturation at 73 °C for 5 min and overnight hybridization at 37 °C in a humidified chamber. Post-hybridization washes included stringent washes at 65 °C and non-stringent washes at room temperature. Slides were counterstained with ProLong Gold Antifade Mountant containing DAPI (Invitrogen).

Fluorescence signals were analyzed using a Zeiss fluorescence microscope (Zeiss, Oberkochen, Germany) equipped with ISIS image analysis software (MetaSystems, Altlussheim, Germany). For each case, 100 intact, non-overlapping interphase nuclei were evaluated. Normal nuclei displayed two fused (yellow) signals, whereas rearranged nuclei exhibited one fused signal and separate red and green signals. Cases were classified as *ALK*-rearranged when > 10% of nuclei demonstrated split signals. This threshold was established empirically using five normal thyroid tissue controls analyzed under the same experimental and analytical conditions used for all study samples. The cutoff value was calculated based on the mean percentage of split signals observed in control tissues plus three standard deviations (mean + 3SD), resulting in a value of 9.8%, which was conservatively rounded to 10% to minimize borderline interpretations.

### ALK Immunohistochemistry (IHC)

ALK protein expression was evaluated by immunohistochemistry as a surrogate marker for ALK fusion-associated chimeric protein expression [30]. Analyses were performed on 3-µm sections of formalin-fixed, paraffin-embedded (FFPE) tissue. Following deparaffinization and rehydration, endogenous peroxidase activity was quenched with 3% hydrogen peroxide for 30 min. Antigen retrieval was carried out in Tris-EDTA buffer (pH 9.0) using a pressure cooker. Non-specific binding was blocked with 5% goat serum in TBST for 1 h at room temperature. Sections were incubated overnight at 4 °C with a rabbit monoclonal anti-ALK antibody (clone D5F3; dilution 1:50; Cell Signaling Technology, Danvers, MA, USA). Immunodetection was performed using the SignalStain^®^ Boost IHC Detection Reagent (HRP, Rabbit; Cell Signaling Technology), according to the manufacturer’s instructions, followed by hematoxylin counterstaining. All nine RT-PCR-positive cases were successfully evaluated by IHC. Commercially available SignalSlide^®^ SU-DHL-1 (positive control) and HeLa (negative control) slides (Cell Signaling Technology) were used in parallel with study samples according to the manufacturer’s recommendations. ALK staining intensity and subcellular distribution were independently assessed by light microscopy by an expert thyroid pathologist blinded to molecular data. Samples were considered positive when ≥ 10% of neoplastic cells demonstrated cytoplasmic staining of any intensity, in accordance with predefined study criteria.

### In silico structural modeling and predicted functional assessment

Predicted protein sequences derived from *STRN::ALK* fusion transcripts were generated using the ExPASy Translate Tool [31]. Open reading frames were selected based on the first in-frame methionine and minimal instability index, prioritizing predicted protein stability. Physicochemical properties, including protein length, molecular weight, theoretical isoelectric point, amino acid composition, grand average of hydropathicity (GRAVY), aliphatic index, and estimated half-life, were computed to support functional inference.

Three-dimensional structural models were generated using the Phyre2 platform based on homology modeling [32]. Predicted structures were compared with canonical *STRN::ALK* fusion proteins to assess domain architecture, integrity of the ALK kinase domain, and potential structural consequences for dimerization and ligand-independent activation. These analyses were used to explore the potential structural and predicted functional consequences of identified fusion variants.

### Statistical Analysis

Statistical analyses were performed using RStudio and GraphPad Prism 10. Categorical variables were compared using Pearson’s Chi-square test or Fisher’s exact test, as appropriate. Continuous variables were analyzed using Student’s *t*-test or one-way ANOVA, as appropriate. When applicable, the Bonferroni correction was applied to adjust for multiple comparisons. A *p*-value < 0.05 was considered statistically significant.

## Results

### Detection of Canonical and Noncanonical *STRN::ALK*-derived Transcripts in Oncocytic Thyroid Neoplasms

**Fig. 1:**
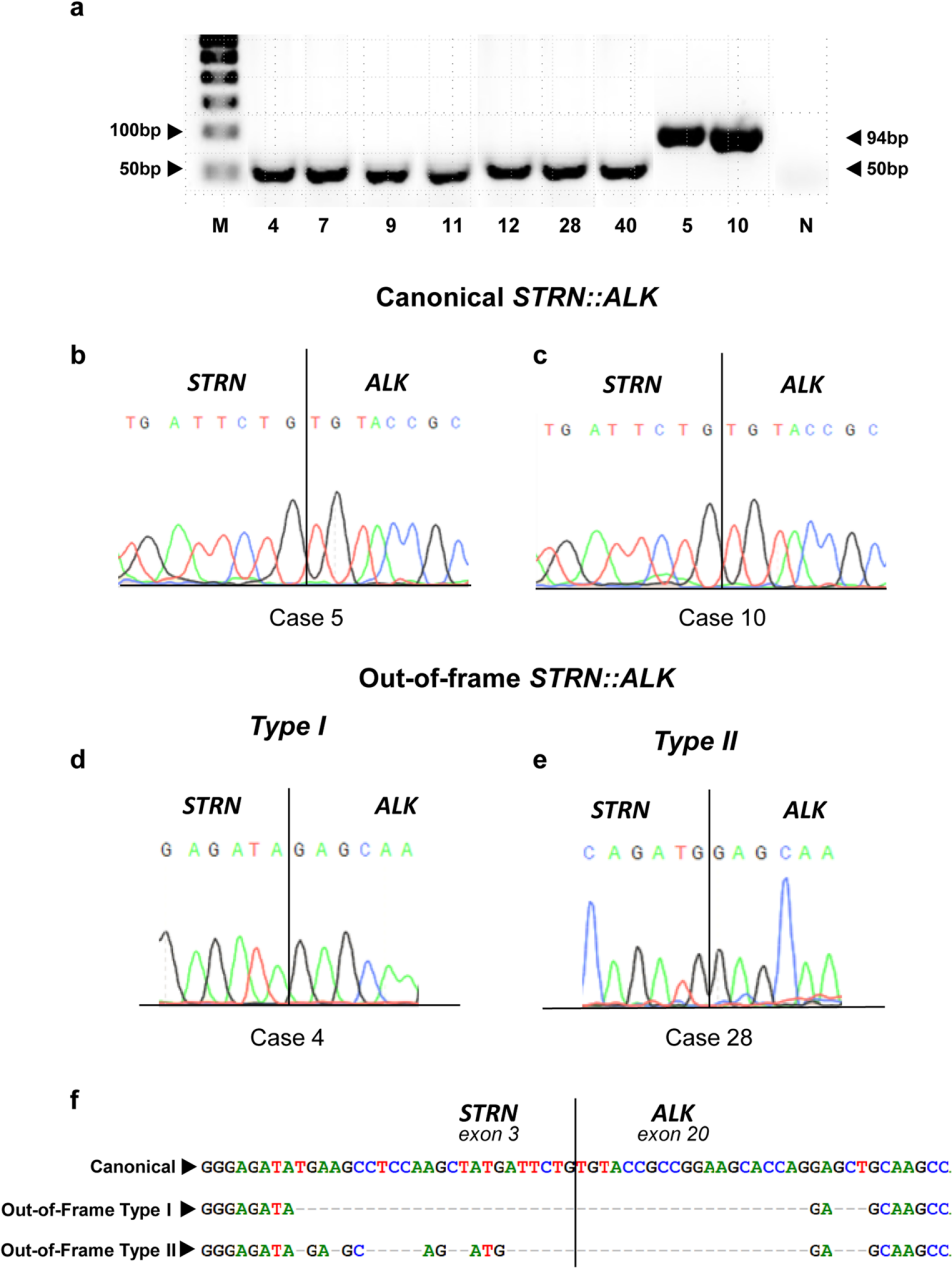
Detection and structural characterization of *STRN::ALK* transcripts in oncocytic thyroid tumors (**a**) RT-PCR screening for *STRN::ALK* transcripts identifies two tumors with the expected 94 bp amplicon corresponding to the canonical fusion, whereas the remaining positive cases exhibit a shorter 50 bp amplicon. M, molecular weight; N, negative control (**b**-**c**) Sanger sequencing electropherograms confirming the canonical in-frame fusion between *STRN* exon 3 and *ALK* exon 20 (**d**-**e**) Representative electropherograms of noncanonical junctions corresponding to Type I and Type II variants observed in tumors with shorter amplicons, demonstrating altered breakpoint usage (**f**) Schematic representation of the canonical *STRN::ALK* fusion alongside two recurrent classes of out-of-frame variants identified in this study. Type I variants are defined by internal deletions leading to frameshifted junctions, whereas Type II variants arise from shifted breakpoints that introduce premature termination codons

RT-PCR screening using a targeted assay for *STRN* exon 3-*ALK* exon 20 junctions identified *STRN::ALK*-derived transcripts in 9 of 56 cases of oncocytic thyroid tumors (16%). Among theses, two OCAs yielded an amplicon of approximately 94 bp, consistent with the expected size of the canonical in-frame *STRN::ALK* fusion. In contrast, the remaining seven positive tumors (five OCAs and two OAs) showed shorter, atypical amplicons of approximately 50 bp (Fig. 1A), suggesting the presence of noncanonical *STRN::ALK*-derived transcripts.

Sanger sequencing confirmed that the 94 bp amplicons identified in 2 of 56 tumors (3.6%) corresponded to *STRN::ALK* junction sequences consistent with a canonical in-frame fusion transcript (Fig. 1B, C).

In the remaining seven of 56 tumors (12.5%), Sanger sequencing of cloned RT-PCR products confirmed *STRN::ALK*-derived sequences retaining the exon 3-exon 20 junction context but showing sequence configurations distinct from the canonical in-frame fusion. These findings were consistent across independent PCR reactions and replicate clones (Fig. 1D, E).

For descriptive purposes, the noncanonical *STRN::ALK*-derived transcripts were categorized based on recurrent sequence patterns observed across independent amplifications and sequenced clones. Two recurrent patterns were identified: Type I variants (Cases 4, 9, 11, and 12), characterized by sequence changes affecting the expected coding frame continuity within the junction region, and Type II variants (Cases 7, 28, and 40), in which sequence configurations were predicted to introduce premature termination codons within the putative fusion transcript (Fig. 1F and Table 2).

**Table 2:** Molecular, Immunohistochemical, and Predicted Structural Features of Canonical and Noncanonical STRN::ALK-positive Oncocytic Thyroid Tumors

Case ID	Diagnosis	RT-PCR product size (bp)	Fusion type	ALK-rearranged nuclei (%)	ALK IHC	ALK kinase-domain features
4	OCA	50	Out-of-frame Type I	15	Negative	Disrupted
5	OCA	94	Canonical	58	Positive	Preserved
7	OCA	50	Out-of-frame Type II	13	Negative	Absent
9	OCA	50	Out-of-frame Type I	12	Negative	Disrupted
10	OCA	94	Canonical	23	Positive	Preserved
11	OCA	50	Out-of-frame Type I	15	Negative	Disrupted
12	OCA	50	Out-of-frame Type I	12	Negative	Disrupted
28	OA	50	Out-of-frame Type II	23	Negative	Absent
40	OA	50	Out-of-frame Type II	35	Negative	Absent

*OCA* Oncocytic Carcinoma of the Thyroid, *OA* Oncocytic Adenoma of the Thyroid

Sequence analysis of the recurrent noncanonical junctions did not identify consistent extended microhomology, direct repeat motifs, or other obvious sequence features that would strongly support systematic polymerase slippage or recurrent RT-PCR artifact generation as the sole explanation for these transcript architectures.

### Genomic Validation Confirms *ALK* Rearrangement Across Canonical and Noncanonical Variants

**Fig. 2:**
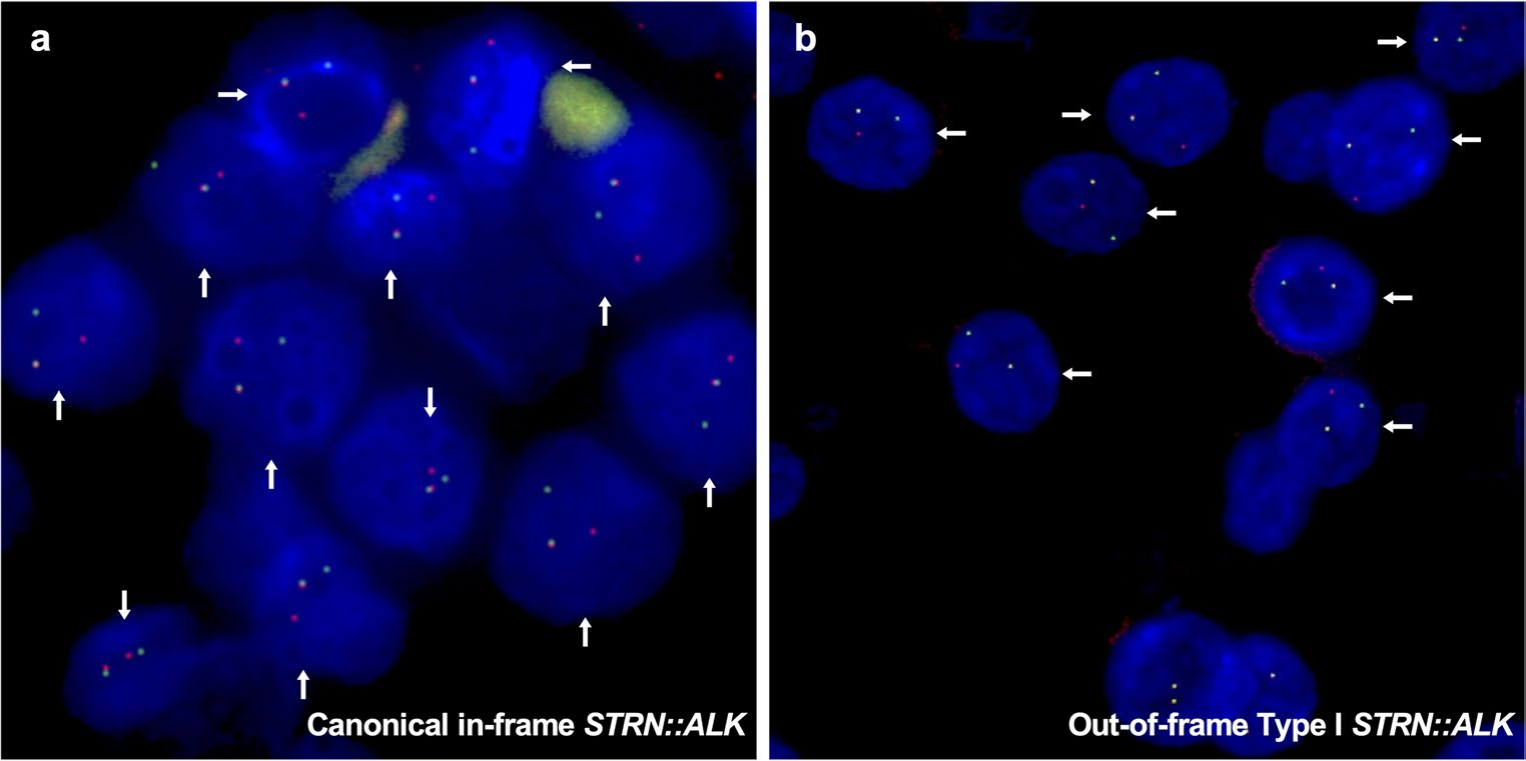
Representative ALK break-apart FISH findings in canonical and noncanonical *STRN::ALK*-positive oncocytic thyroid tumors (**a**) Representative oncocytic thyroid carcinoma harboring a canonical in-frame *STRN*::*ALK* fusion demonstrating split ALK break-apart signals consistent with genomic rearrangement (**b**) Representative oncocytic thyroid tumor harboring a noncanonical out-of-frame *STRN*::*ALK* variant also demonstrating split ALK break-apart signals. White arrows indicate nuclei with rearranged ALK signals. Original magnification: ×1000

Break-apart FISH confirmed *ALK* rearrangement in all nine RT-PCR-positive neoplasms, with split signals detected in 12 to 58% of nuclei. ALK rearrangement signals were identified in both canonical in-frame fusion cases (Case 5, Fig. 2A; Case 10, Supplementary Fig. 1) and out-of-frame variants (Case 4, Fig. 2B; cases 7, 9, 11, 12, 28, and 40, Supplementary Fig. 1).

### ALK Protein Expression Is Restricted to Canonical *STRN::ALK* Oncocytic Thyroid Carcinomas

**Fig. 3:**
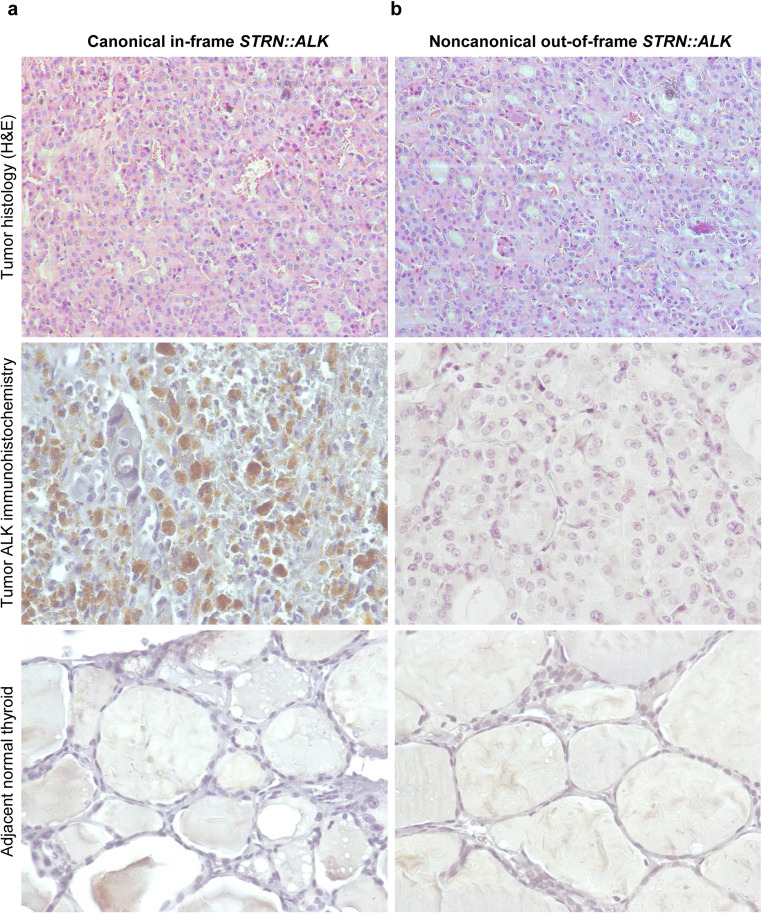
Histologic and immunohistochemical features of canonical and noncanonical *STRN::ALK*-positive oncocytic thyroid tumors (**a**) Representative oncocytic thyroid carcinoma harboring a canonical in-frame *STRN*::*ALK* fusion. Upper panel: hematoxylin and eosin (H&E) staining demonstrating typical oncocytic morphology with abundant eosinophilic cytoplasm. Middle panel: diffuse cytoplasmic ALK immunoreactivity in tumor cells. Lower panel: adjacent normal thyroid tissue lacking ALK expression, serving as an internal negative control (**b**) Representative oncocytic thyroid tumor harboring a noncanonical out-of-frame *STRN*::*ALK* variant. Upper panel: H&E staining confirming oncocytic morphology. Middle panel: absence of ALK immunoreactivity despite confirmed genomic rearrangement by FISH. Lower panel: adjacent normal thyroid tissue negative for ALK expression. H&E images were obtained at ×200 magnification; immunohistochemical images were obtained at ×400 magnification

ALK protein expression was exclusively observed in tumors harboring canonical in-frame fusions. Specifically, diffuse cytoplasmic ALK immunoreactivity was observed exclusively in oncocytic carcinomas with canonical *STRN::ALK* fusions (Case 5, Fig. 3A; Case 10, Supplementary Fig. 2A). In contrast, all neoplasms carrying noncanonical out-of-frame variants lacked detectable ALK expression, with no focal, weak, or equivocal tumor staining observed in any case (Case 4, Fig. 3B; Cases 7, 9, 11, 12, 28, and 40, Supplementary Fig. 2B and C). Matched adjacent normal thyroid tissue was consistently negative for ALK expression.

### Structural Modeling Suggests Disruption of Kinase-domain Features in Out-of-frame Variants

**Fig. 4:**
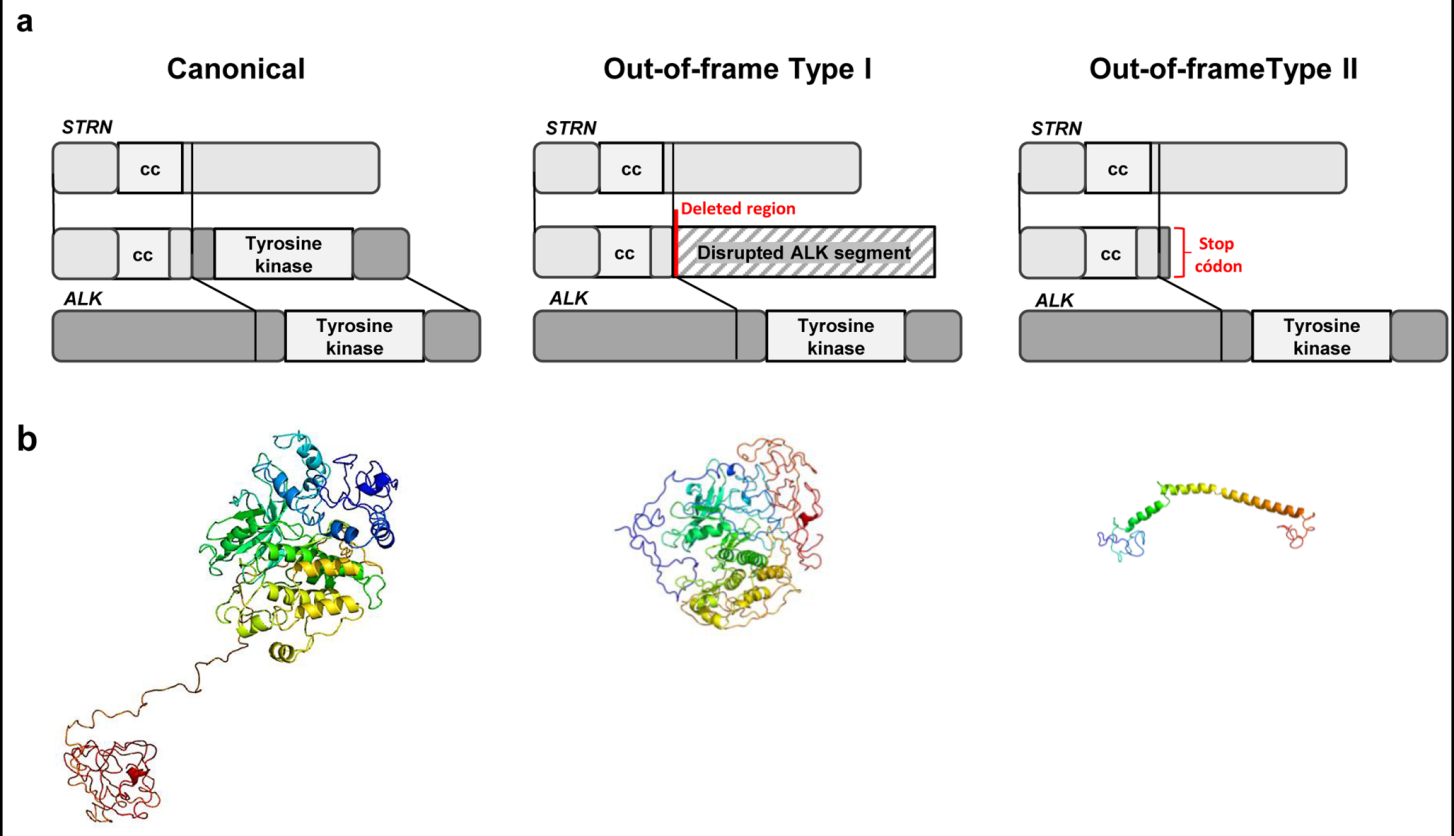
Structural organization and in silico modeling of canonical and noncanonical *STRN::ALK *fusion variants identified in oncocytic thyroid tumors (**a**) Schematic representation of canonical and noncanonical *STRN*::*ALK* fusion architectures detected in this study. The canonical variant corresponds to the expected in-frame *STRN* exon 3-*ALK* exon 20 fusion, preserving the ALK kinase domain. Noncanonical variants include structurally altered out-of-frame transcripts characterized by internal deletions and/or premature stop codons predicted to disrupt preservation of kinase-domain features (**b**) In silico structural modeling of representative canonical and noncanonical fusion products. Canonical fusions retained overall structural organization consistent with preservation of ALK kinase-domain features, whereas noncanonical variants demonstrated partial structural disruption (Type I) or severe truncation with absence of recognizable kinase-domain organization (Type II). Structural models are displayed at proportional relative scale to illustrate the predicted truncation of noncanonical variants

Homology-based structural modeling suggested that the canonical *STRN::ALK* fusion retains an intact ALK kinase domain architecture, consistent with preservation of key structural elements of the kinase core. In contrast, all out-of-frame variants exhibited structural disruption and segregated into two recurrent architectures. Type I variants (Cases 4, 9, 11, and 12) were predicted to encode proteins of approximately 685 amino acids with internal deletions affecting continuity of the ALK-derived region and resulting in partial preservation of kinase-related sequences together with increased predicted structural disorder. Type II variants (Cases 7, 28, and 40) were predicted to generate severely truncated proteins of approximately 145 amino acids due to premature termination, consisting predominantly of STRN-derived sequences and lacking ALK coding regions (Fig. 4A and B).

Structural similarity analysis was concordant with these observations. Canonical fusions were compatible with preservation of overall kinase-domain organization, whereas noncanonical variants demonstrated predicted structural disruption in Type I variants and absence of kinase-domain features in Type II variants. Collectively, these findings suggest differential preservation of kinase-domain organization across fusion architectures; however, these conclusions are based on in silico modeling and represent structural inference rather than direct experimental evidence of kinase activity.

Structural modeling metrics, including predicted kinase-domain preservation, Phyre2 confidence scores, and template identity metrics, are summarized in Table 3.

**Table 3:** Structural modeling and physicochemical properties of predicted protein variants

Parameter	Canonical	Type I	Type II
Length (aminoacids)	700	685	145
% residues > 90% confidence	55%	44%	32%
Main template	Kinase (ALK-like)	Kinase-like (partial)	STRN (non-kinase)
Structural interpretation	Kinase domain preserved	Disruption of kinase domain	No kinase domain retained
Molecular weight	77524.51	75651.35	15951.02
Theoretical pI	7.87	7.23	9.17
Total number of negatively charged residues (Asp + Glu)	78	77	17
Total number of positively charged residues (Arg + Lys)	80	77	21
Formula	C3441H5396N962O1014S33	C3357H5267N937O992S32	C702H1103N213O206S4
Total number of atoms	10,846	10,585	2228
The estimated half-life	30 h	30 h	30 h
The instability index	49.11	47.53	29.34
Aliphatic index	73.90	74.53	70.28
Grand average of hydropathicity (GRAVY)	−0.488	−0.469	−0.610
Cases presenting this protein conformation	5 and 10	4, 9, 11, and 12	7, 28, and 40

### Canonical *STRN::ALK* fusions Were Observed only in Malignant Oncocytic Tumors

Canonical *STRN::ALK* fusions were observed exclusively in oncocytic carcinomas (OCAs), representing 2 of 56 tumors overall (3.6%) and 2 of 27 OCAs (7.4%) within the cohort. No significant differences in baseline clinicopathological features, including patient sex, age, tumor size, or other available indicators of tumor aggressiveness, were observed between tumors harboring canonical in-frame fusions and those classified as noncanonical *STRN::ALK*-derived variants.

## Discussion

To our knowledge, this is the first study to characterize *STRN* exon 3-*ALK* exon 20-derived transcripts in a dedicated cohort of oncocytic tumors of the thyroid using an integrated RNA-, genomic-, and protein-based approach. Our findings indicate that *STRN*::*ALK*-positive oncocytic thyroid neoplasms comprise distinct transcript architectures associated with divergent ALK protein expression patterns. Although genomic *ALK* rearrangement was confirmed by FISH in all transcript-positive tumors, detectable ALK protein expression was restricted to canonical in-frame fusions, whereas noncanonical variants remained consistently immunonegative. These findings suggest that fusion architecture may influence preservation of kinase-domain organization and ALK protein expression, supporting cautious interpretation of ALK rearrangements in oncocytic thyroid neoplasms.

Importantly, both canonical and noncanonical *STRN::ALK*-derived transcripts were identified in tumors demonstrating genomic ALK rearrangement by FISH, indicating that genomic-level ALK disruption alone may not predict downstream protein expression. The restriction of ALK immunoreactivity to tumors harboring canonical in-frame fusions, together with the predicted preservation of kinase-domain organization in these cases, suggests that only a subset of ALK-rearranged oncocytic thyroid neoplasms generate fusion transcripts compatible with stable ALK protein expression. These observations support the existence of a hierarchical relationship across genomic, transcript, and protein levels in which the biological consequences of ALK rearrangement may depend on fusion architecture rather than rearrangement status alone.

The recurrent detection of noncanonical variants across independent PCR amplification, cloning, and sequencing experiments supports the reproducibility of these findings under the experimental conditions applied in this study. In addition, all noncanonical transcripts were consistently associated with FISH-confirmed ALK rearrangement. Together, these observations suggest that the identified variants are unlikely to represent purely stochastic technical artifacts. Nevertheless, given the use of archival FFPE material, short-amplicon RT-PCR, and cloning-based Sanger sequencing, alternative explanations, including RNA fragmentation, template switching, preferential amplification of shorter products, and recurrent sequence-dependent PCR artifacts, cannot be definitively excluded and may contribute to the observed diversity of junction structures.

Structural modeling indicated that canonical fusions preserve ALK kinase-domain architecture, whereas noncanonical variants are associated with predicted structural disruption. However, these in silico predictions should be interpreted as supportive but not definitive evidence of functional integrity. Additional biological considerations further complicate the interpretation of these variants. Out-of-frame transcripts containing premature termination codons, particularly those classified as Type II variants, may be subject to nonsense-mediated mRNA decay, potentially limiting production of stable protein products and raising the possibility that some predicted truncated proteins are not expressed in vivo. Even in the absence of preserved kinase-domain features, *STRN::ALK* fusion products retaining STRN-derived regions could theoretically exert biological effects independent of ALK signaling. In particular, potential ALK-independent mechanisms, including disruption of STRIPAK complex assembly and PP2A-associated signaling pathways, may be considered given the scaffolding role of STRN (striatin) in this network. Although speculative, these considerations further support interpreting noncanonical variants as rearrangements of uncertain biological significance.

Consistent with this, discordance between FISH and IHC likely reflects differences in fusion architecture rather than a single uniform ALK alteration, as also reported in other tumor types, including thyroid neoplasms [18, 22, 33, 34]. In oncocytic thyroid tumors, this is particularly relevant in the context of marked mitochondrial accumulation and genomic instability, which may promote structurally diverse rearrangements with variable biological consequences [5, 6].

Canonical *STRN*::*ALK* fusions were identified exclusively in oncocytic thyroid carcinomas within this cohort, whereas noncanonical variants were observed in both carcinomas and adenomas. Given the limited number of canonical cases, this observation should be interpreted cautiously and considered descriptive of the present cohort rather than evidence of a tumor-type-specific biological distribution. Although no significant clinicopathological differences were identified between canonical and noncanonical groups, these comparisons remain substantially underpowered and should not be interpreted as evidence of biological equivalence. Nevertheless, the restriction of canonical in-frame fusions to malignant tumors raises the possibility that structurally intact rearrangements may be more likely to generate biologically relevant ALK protein products.

The apparent rarity and heterogeneity of these alterations, together with differences in cohort composition, may also account for the absence of ALK rearrangements in two large genomic profiling studies of oncocytic thyroid carcinomas by Gopal et al. and Ganly et al. [5, 6], despite isolated reports of ALK-rearranged oncocytic thyroid tumors confirmed by FISH and immunohistochemistry [26, 27]. In the present cohort, although *STRN*::*ALK*-derived transcripts were detected in 9 of 56 tumors (16%) using this targeted assay, only 2 of 56 cases (3.6%) harbored canonical in-frame fusions associated with detectable ALK protein expression. These observations suggest that biologically relevant canonical *STRN*::*ALK* alterations likely represent a substantially smaller subset of oncocytic thyroid neoplasms than overall transcript detection rates alone might imply, while structurally atypical transcripts may contribute to apparent variability across studies. Differences in cohort composition, assay design, and sensitivity for detection of structurally altered transcripts may also contribute to these discrepancies. Together, these observations suggest a potential role for integrated molecular and protein-based assessment in the interpretation of ALK status and stratification of cases for future investigation of ALK-targeted therapies.

This study has limitations inherent to its design. The targeted RT-PCR approach was restricted to a single *STRN* exon 3-*ALK* exon 20 junction configuration and therefore did not capture the full spectrum of ALK fusion diversity. Accordingly, *ALK* fusions involving alternative breakpoints within *STRN* or *ALK*, as well as rearrangements involving non-STRN fusion partners, may have been missed.

In addition, RNA degradation in archival FFPE material may have contributed to false-negative RT-PCR results despite the potential presence of underlying genomic ALK rearrangements. The possibility of RT-PCR-negative/FISH-positive discordant cases cannot be excluded, as FISH analysis was restricted to RT-PCR-positive tumors, introducing a degree of ascertainment bias. Consequently, the reported detection rate reflects assay performance rather than the true prevalence of ALK rearrangements in oncocytic thyroid tumors.

FFPE-derived RNA is inherently fragmented, increasing susceptibility to artifacts in short-amplicon assays. Although sequence inspection did not identify consistent microhomology, direct repeats, or other features suggestive of polymerase slippage, sequence-dependent artifacts cannot be excluded. Moreover, the analyzed products derive from RT-PCR-amplified, cloned, and sequenced fragments and therefore represent assay-derived transcript segments rather than full-length fusion transcripts. Thus, the data represent transcript fragments generated under assay conditions rather than complete fusion structures.

Cloning-based Sanger sequencing provides limited resolution of transcript complexity and is subject to amplification bias, which may preferentially enrich specific amplicons while underrepresenting low-abundance variants. Consequently, functional interpretation based on immunohistochemistry and in silico structural modeling should be considered preliminary, as these methods do not provide direct experimental validation of biological activity. Orthogonal approaches, including long-read RNA sequencing, comprehensive genomic profiling, and functional assays, are required to define transcript architecture and biological relevance. Notably, the recurrent detection of structurally distinct *STRN::ALK*-derived transcripts across independent cases supports the robustness of the findings and argues against isolated technical artifacts.

Finally, although all cases underwent centralized expert histopathological review according to WHO 2022 criteria, formal inter-observer agreement analysis was not performed, and the modest sample size limited clinicopathological correlations. Future studies incorporating larger, independent cohorts with integrated RNA-based and functional validation will be essential to confirm and extend these findings, particularly regarding the biological significance of noncanonical variants.

In summary, *STRN::ALK* rearrangements in oncocytic thyroid tumors appear to comprise two categories with distinct structural and predicted functional characteristics: rare canonical in-frame fusions associated with detectable ALK protein expression and predicted preservation of kinase-domain features, and more frequent noncanonical variants that are structurally altered and of uncertain biological relevance. Collectively, these findings suggest that detection of *STRN::ALK*-derived transcripts alone may not uniformly reflect biologically relevant fusion protein expression, supporting the importance of integrative molecular interpretation when evaluating *STRN::ALK*-positive oncocytic thyroid tumors.

## Supplementary Information

Below is the link to the electronic supplementary material.

Supplementary Material 1

ESM 2 (PNG 862 KB)

High Rsolution Image (TIF 4.20 MB)

ESM 3 (PNG 5.01 MB)

High Rsolution Image (TIF 10.9 MB)

## Data Availability

All data supporting the findings of this study are available within the paper and its Supplementary Information.
