# Supplementary material for: Distinct ALK Expression Patterns Are Associated with Canonical and Noncanonical STRN::ALK Transcript Architectures in Oncocytic Thyroid Neoplasms
Source: Endocr Pathol. 2026 Jul 2;37(1):27. doi: 10.1007/s12022-026-09924-0 (PMC13328310; doi:10.1007/s12022-026-09924-0)
Supplement: Supplementary file 1 — Supplementary Material 1 [file 12022_2026_9924_MOESM1_ESM.docx]

**Supplementary Material**

**Supplementary Figures**

**Supplementary Figure 1. *ALK break-apart FISH analysis in STRN::ALK-positive oncocytic thyroid tumors.*.**

**
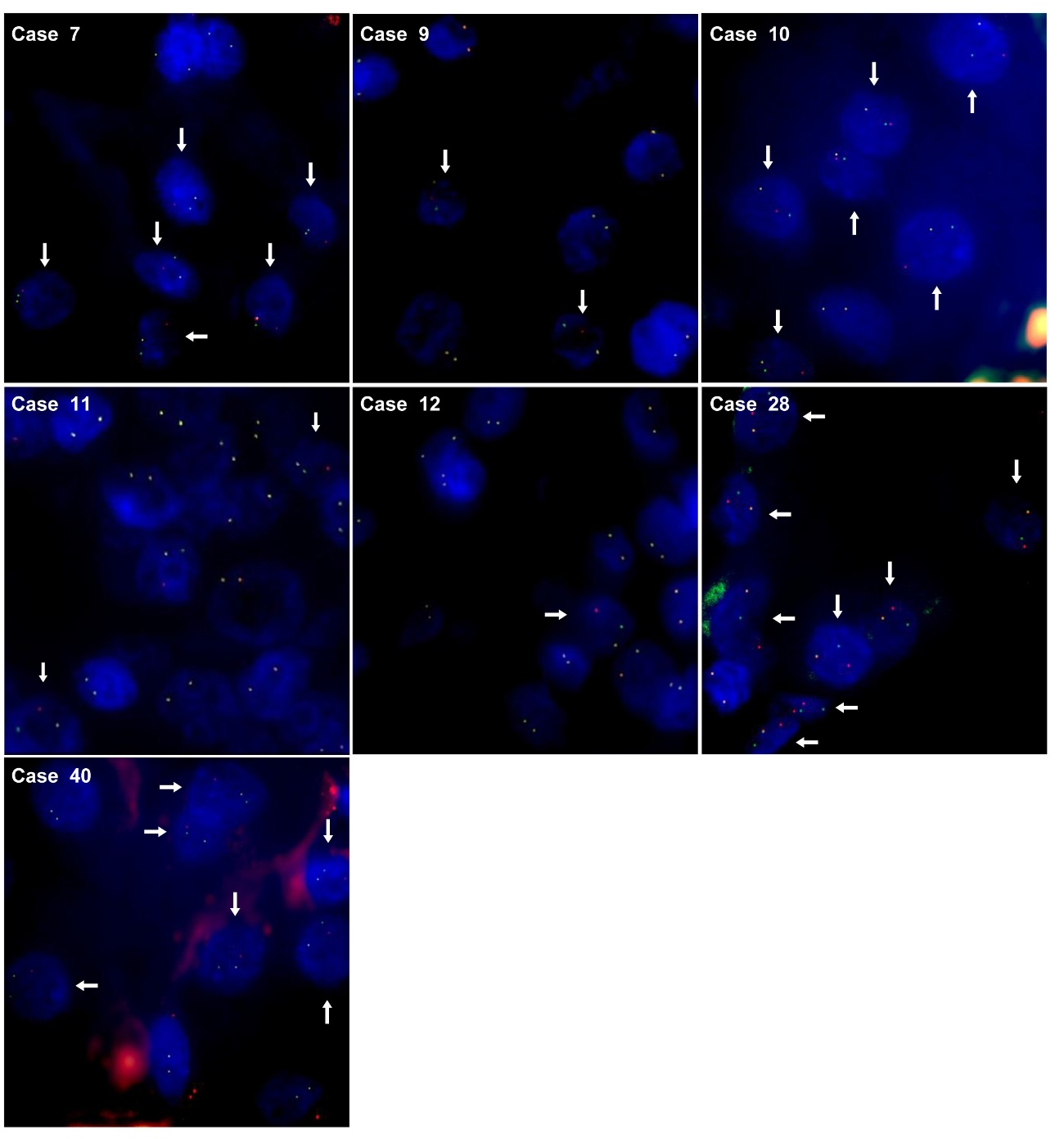
**

Representative dual-color ALK break-apart FISH images from all *STRN*::*ALK*-positive oncocytic thyroid tumors included in this study. White arrows indicate nuclei demonstrating split red and green signals consistent with ALK rearrangement, whereas fused yellow signals correspond to intact ALK alleles. Case 10 harbors the canonical in-frame *STRN*::*ALK* fusion, whereas Cases 7, 9, 11, 12, 28, and 40 correspond to noncanonical out-of-frame variants.

**Supplementary Figure 2. ALK immunohistochemistry in *STRN*::*ALK*-positive oncocytic thyroid tumors (original magnification ×200).**


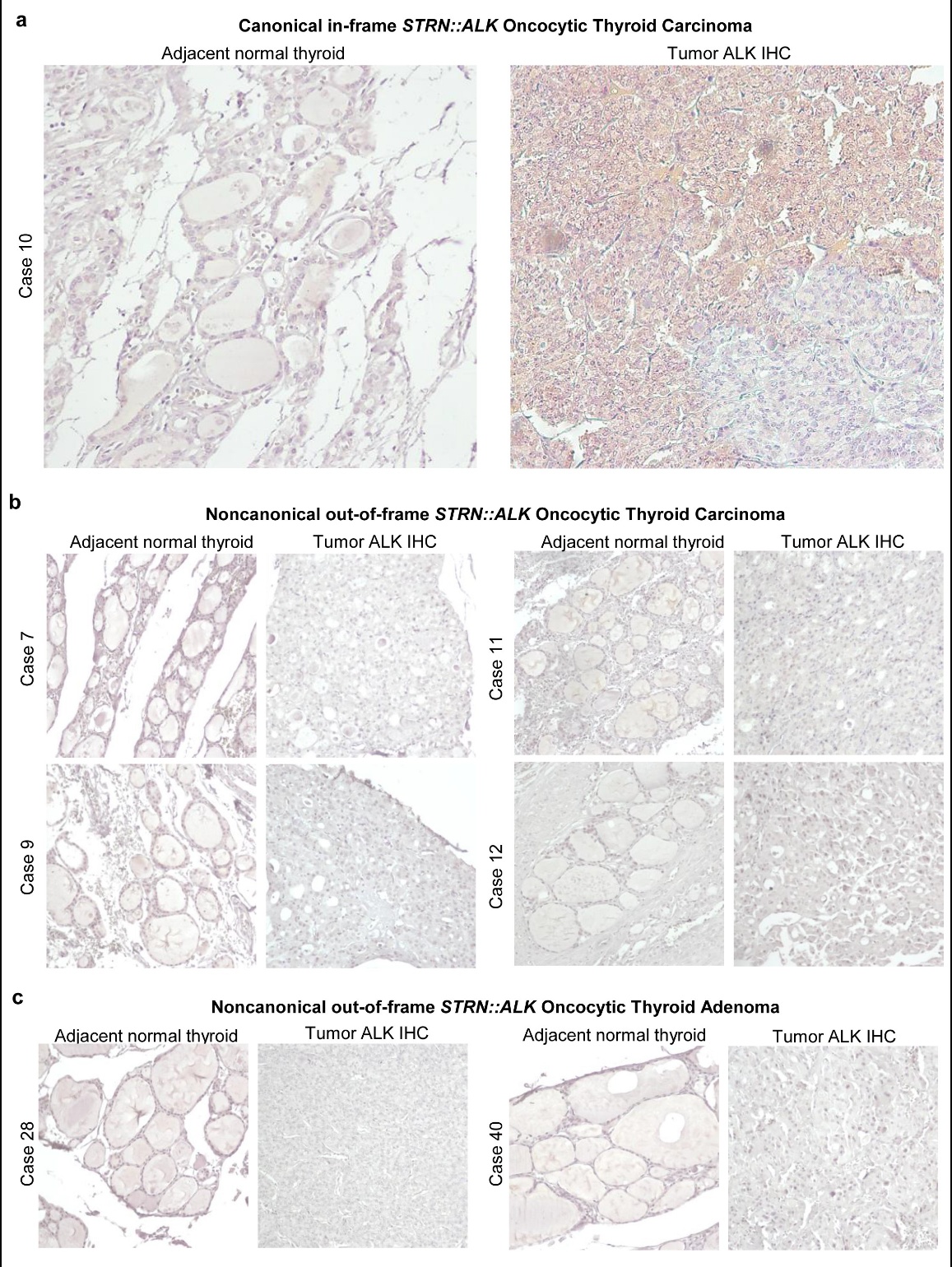


(A) Canonical in-frame *STRN*::*ALK* oncocytic thyroid carcinoma showing diffuse cytoplasmic ALK staining in tumor cells, with matched adjacent normal thyroid tissue negative for ALK expression.
(B) Noncanonical out-of-frame *STRN*::*ALK* oncocytic thyroid carcinomas showing no detectable ALK expression in tumor cells.
(C) Noncanonical out-of-frame *STRN*::*ALK* oncocytic thyroid adenomas showing no detectable ALK expression in tumor cells. Adjacent normal thyroid tissue was negative in all cases.
