# Supplementary figures and images for: Distinct ALK Expression Patterns Are Associated with Canonical and Noncanonical STRN::ALK Transcript Architectures in Oncocytic Thyroid Neoplasms
Source: Endocr Pathol. 2026 Jul 2;37(1):27. doi: 10.1007/s12022-026-09924-0 (PMC13328310; doi:10.1007/s12022-026-09924-0)

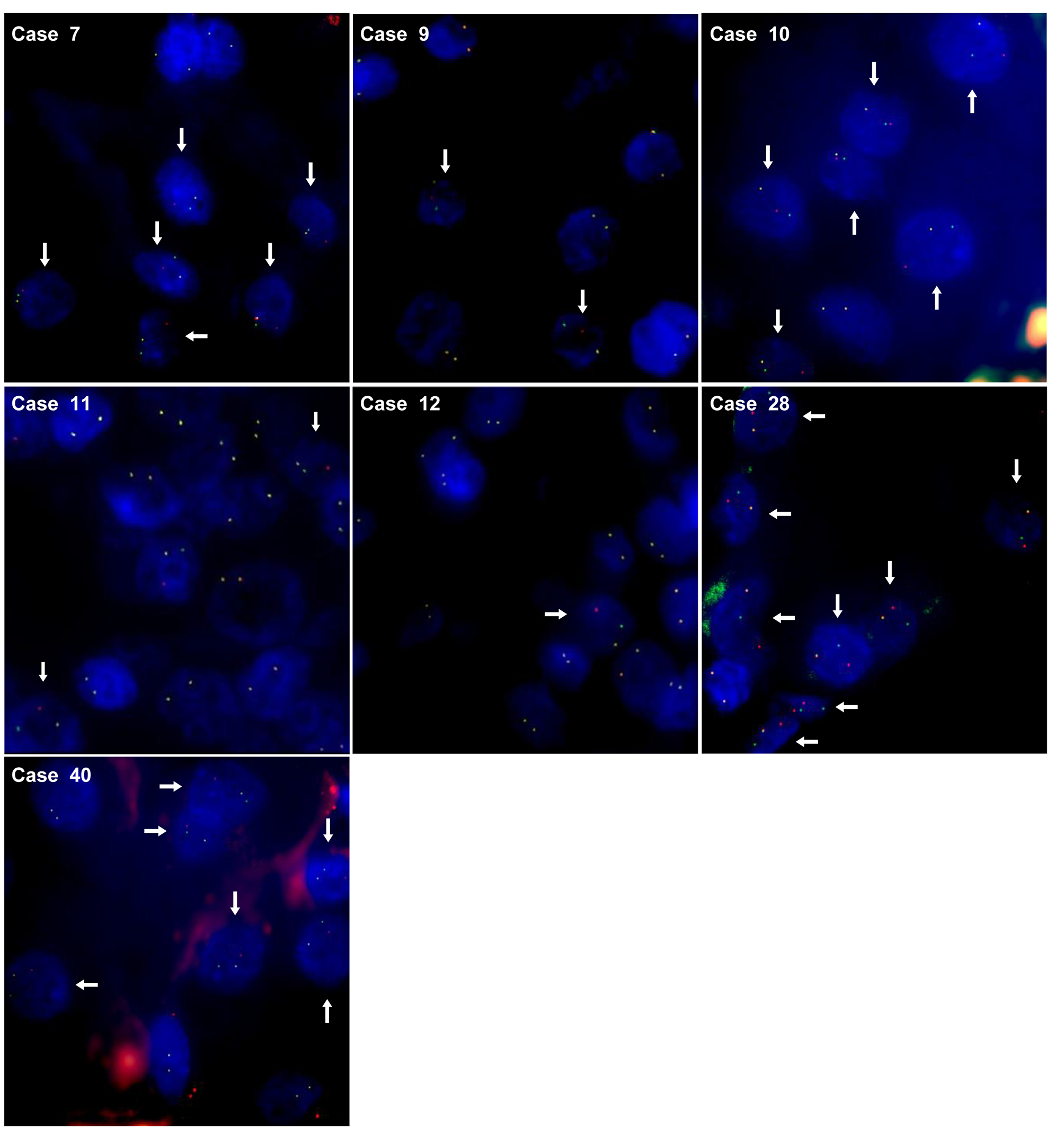

Supplement: Supplementary file 2 — (PNG 862 KB) [file 12022_2026_9924_Fig5_ESM.png]

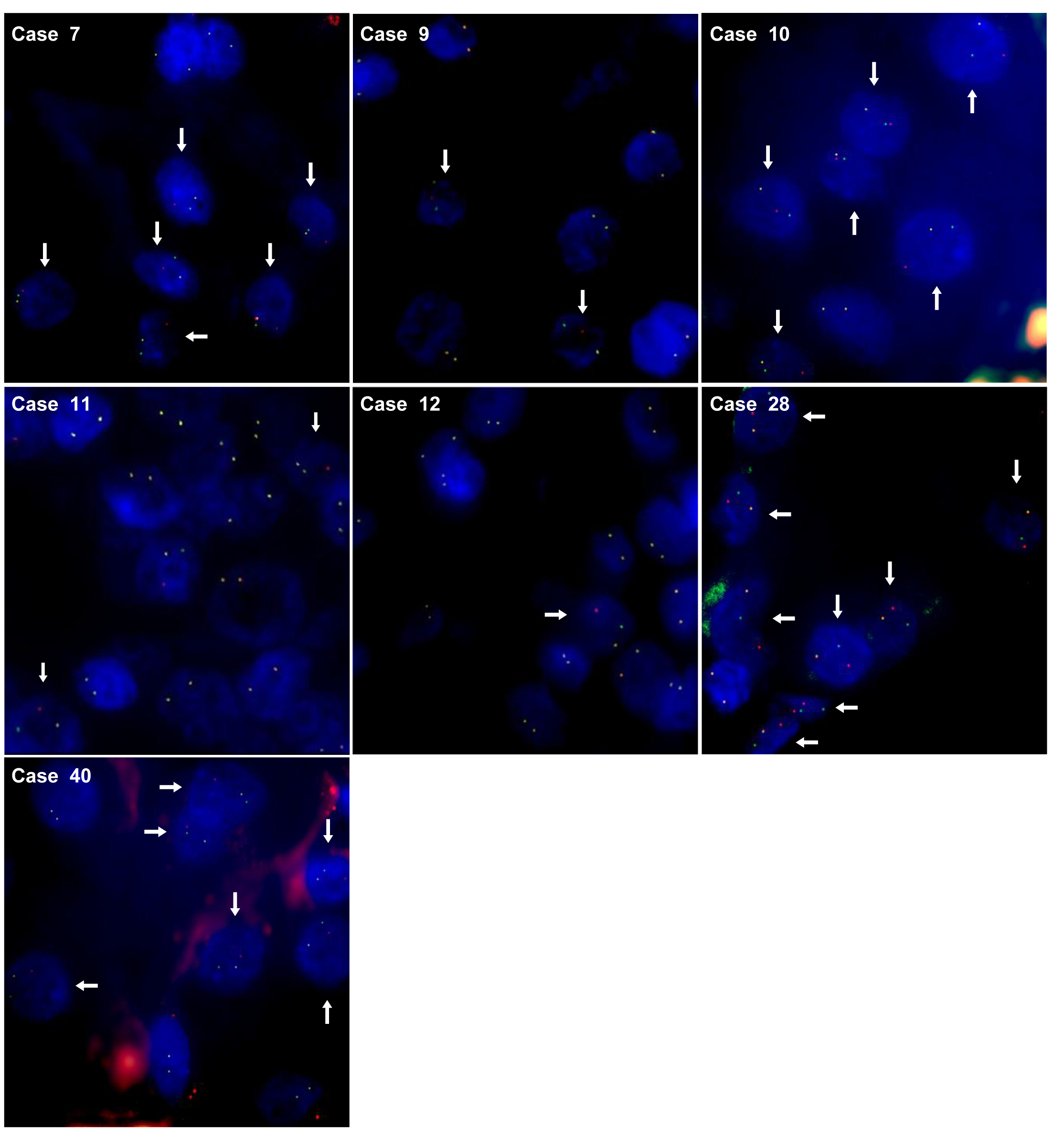

Supplement: Supplementary file 3 — High Rsolution Image (TIF 4.20 MB) [file 12022_2026_9924_MOESM2_ESM.tif]

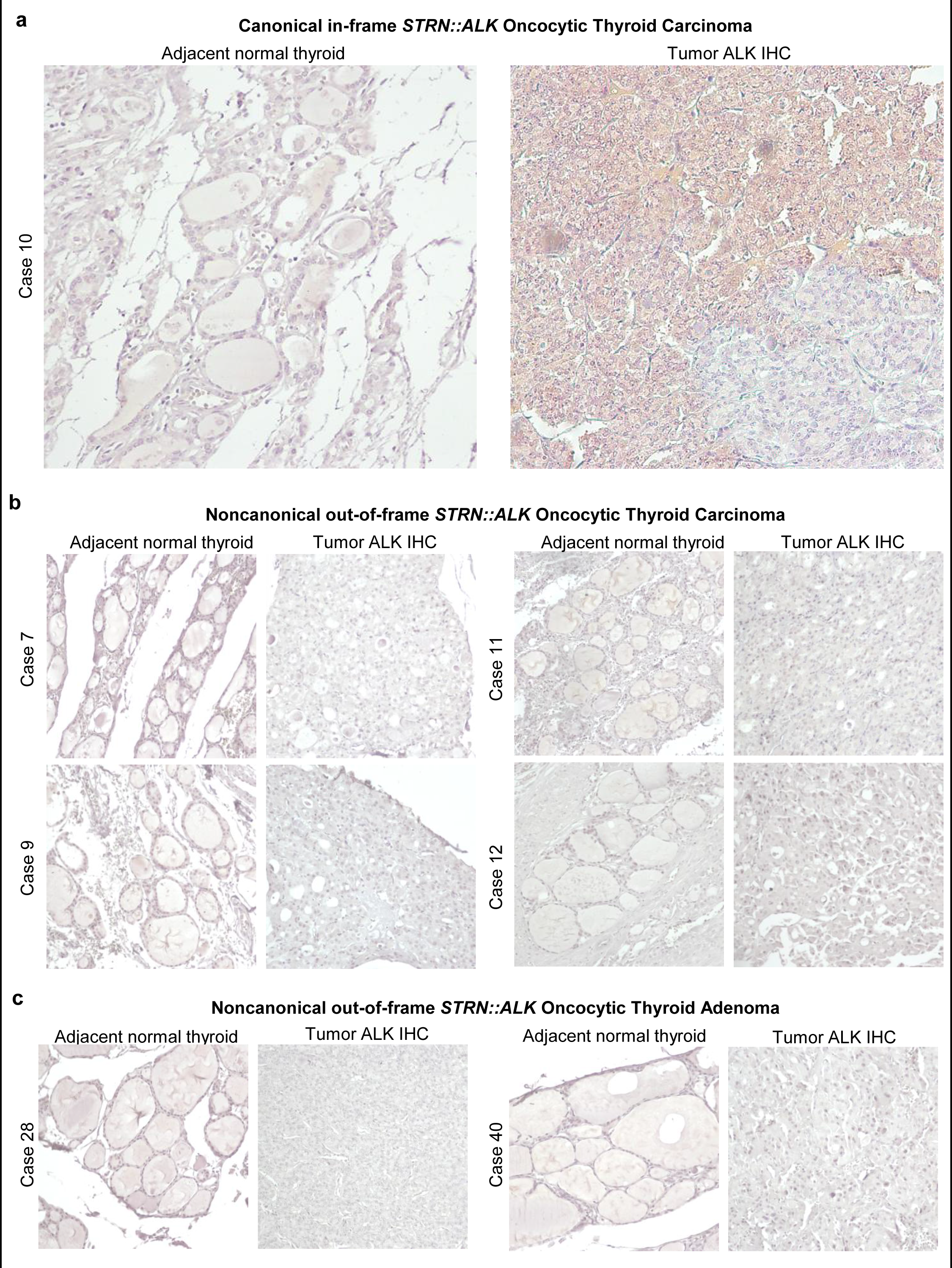

Supplement: Supplementary file 4 — (PNG 5.01 MB) [file 12022_2026_9924_Fig6_ESM.png]

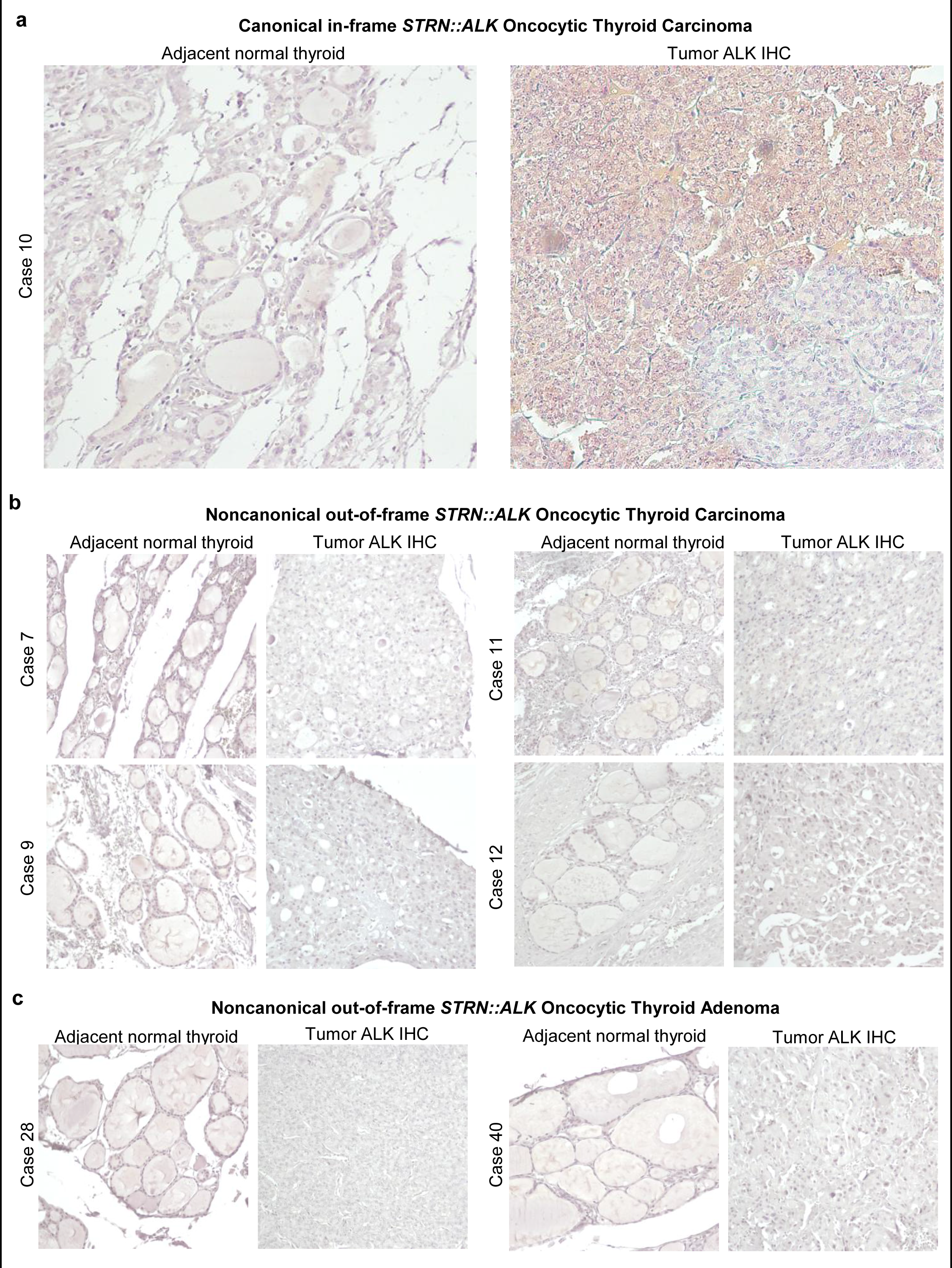

Supplement: Supplementary file 5 — High Rsolution Image (TIF 10.9 MB) [file 12022_2026_9924_MOESM3_ESM.tif]
